# Albumin‐To‐Creatinine Ratio Underestimates True 24‐Hour Albuminuria in Obesity: Clinical Relevance for Vascular Risk Stratification

**DOI:** 10.1002/dmrr.70064

**Published:** 2025-06-25

**Authors:** D. Moriconi, M. Nannipieri, M. Jadoon, A. Solini, R. M. Bruno

**Affiliations:** ^1^ Department of Clinical and Experimental Medicine University of Pisa Pisa Italy; ^2^ Department of Information Engineering University of Pisa Pisa Italy; ^3^ INSERM U970 Team 7 Paris Cardiovascular Research Centre—PARCC Universitè Paris‐Cité Paris France; ^4^ Department of Surgical Medical Molecular and Critical Area Pathology University of Pisa Pisa Italy

**Keywords:** albuminuria, arterial stiffness, endothelial dysfunction, microalbuminuria, obesity

## Abstract

**Aims:**

Albuminuria is a recognized marker of endothelial dysfunction and early cardiovascular risk. The albumin‐to‐creatinine ratio (ACR) is widely used to estimate urinary albumin excretion, but in individuals with high fat‐free mass (FFM), such as those with obesity, elevated urinary creatinine may lead to underestimation of albuminuria. We aimed to investigate the concordance between ACR and 24‐h urinary albumin excretion (UAE) in adults with obesity and examine whether discrepancies affect the detection of vascular dysfunction.

**Methods:**

140 individuals affected by severe obesity were enrolled. Albuminuria was assessed using both spot ACR and 24‐h UAE. A subgroup of 70 participants underwent vascular testing, including carotid‐femoral pulse wave velocity (cf‐PWV) and allometrically scaled flow‐mediated dilation (FMD). Multivariable linear models were used to evaluate associations between albuminuria markers and vascular parameters, adjusting for age, sex, blood pressure, and HbA1c.

**Results:**

24h‐UAE ≥ 30 mg was more frequent in males (35%) than females (20%), while ACR ≥ 30 mg/g showed no sex difference; 21 individuals (15%) exhibited elevated 24 h‐UAE (≥ 30 mg/24 h) despite a normal ACR (< 30 mg/g), a discordant pattern predominantly observed in males with high FFM and urinary creatinine levels. Interestingly, both ACR and UAE were independently associated with reduced FMD (st.*β* = −0.27 and −0.24; *p* < 0.05). No sex‐based interactions were observed in the vascular models.

**Conclusions:**

In individuals with obesity, ACR may underestimate albuminuria, especially in males. Despite this, both markers are associated with early endothelial dysfunction. UAE may provide added value in cardiovascular risk stratification where ACR underperforms.

## 1 Introduction

An increased urinary albumin excretion rate is an important early indicator of kidney damage and a significant predictor of cardiovascular morbidity and mortality [1, 2]. Moderately increased albuminuria, historically termed as ‘microalbuminuria’ and defined as 30 mg/daily, reflects both glomerular injury and systemic endothelial dysfunction [3, 4], and its presence has been associated with increased arterial stiffness and impaired vascular reactivity, even in individuals without overt kidney disease [5, 6].

Among the available measures of albuminuria, the albumin‐to‐creatinine ratio (ACR) obtained from a spot urine sample is widely used in clinical and epidemiological settings due to its convenience and relatively good correlation with 24‐h urinary albumin excretion (24 h‐UAE) [7, 8, 9].

However, ACR is influenced by urinary creatinine concentration, which in turn is strongly determined by muscle mass [10, 11]. Individuals with high fat‐free mass, such as those with obesity, tend to have higher urinary creatinine excretion, which may lead to an underestimation of albuminuria when assessed through ACR [12].

Most clinical guidelines, including the KDIGO recommendations [13, 14], endorse a fixed ACR threshold of 30 mg/g for the diagnosis of moderately increased albuminuria. However, in the light of what reported above, recent evidence suggests that such cutoff may be suboptimal in populations with altered creatinine kinetics such as individuals with severe obesity [12].

Twenty four h‐UAE, while less easy to obtain, represents a true quantification of albumin excretion, fully independent of creatinine levels, and may therefore be of greater utility in patients with severe obesity, where a discrepancy between ACR and 24 h‐UAE may arise, potentially affecting clinical interpretation and risk stratification. However, it remains unclear whether such discordance has functional implications in terms of vascular health.

It is also worth considering that obesity itself is a condition characterised by multiple pathophysiological mechanisms, such as inflammation, insulin resistance, and altered haemodynamics, which collectively predispose individuals to early vascular damage [15, 16]. Understanding whether standard albuminuria markers adequately reflect this risk in obese individuals is therefore clinically relevant.

The aim of this study was to compare ACR and 24 h‐UAE in a cohort of obese adults undergoing pre‐bariatric surgery evaluation, and to investigate whether discrepancies between these measures are associated with early vascular impairment, as assessed by pulse wave velocity (PWV) and flow‐mediated dilation (FMD). A secondary objective was to explore the potential role of urinary creatinine excretion and body composition in modulating the diagnostic performance of ACR in this context.

## 2 Materials and Methods

This cross‐sectional study included 130 consecutive adult patients referred to the Outpatient Clinic for metabolic diseases of the University Hospital in Pisa for pre‐surgery evaluation prior to bariatric procedure. Exclusion criteria included the presence of known glomerular disease, urinary tract infection, cancer, or heart/liver failure.

Patients were screened for albuminuria using both 24 h‐UAE and ACR; those with 24 h‐UAE ≥ 30 mg/24 h and/or ACR ≥ 30 mg/g were all invited to undergo vascular assessment including carotid‐femoral pulse wave velocity (PWV) and brachial flow‐mediated dilation (FMD). To provide a comparative reference, an equal number of normoalbuminuric subjects (24 h‐UAE < 30 mg/24 h and ACR < 30 mg/g) was also included in the vascular study. This enrichment‐based selection strategy aimed to maximise the likelihood of detecting early subclinical vascular alterations in at‐risk individuals while preserving a physiological contrast through the inclusion of normoalbuminuric controls.

In patients treated with ACE inhibitors or angiotensin receptor blockers (ARBs), therapy was temporarily discontinued at least 72 h prior to urine collection, replacing it with calcium‐channel blockers. All enrolled participants with T2D were treated with metformin, either alone or in combination with insulin. None were receiving sodium‐glucose cotransporter‐2 (SGLT2) inhibitors or glucagon‐like peptide‐1 receptor agonists (GLP‐1 RAs) at the time of the study.

The study was conducted in accordance with the Declaration of Helsinki and was approved by the local Institutional Ethics Committee. Informed written consent was obtained from all participants prior to enrolment.

### 2.1 Experimental Session

All participants had a detailed medical history and a complete physical examination. Office blood pressure was measured at the brachial level with the patients resting in the supine position for at least 10 min, using an automatic oscillometric device (OMRON‐705IT, Omron Corporation, Kyoto, Japan), with a suitable adult‐size cuff according to arm circumference. Venous blood samples were collected after an overnight fast for standard biochemistry. On the first study day, after an overnight fast, venous blood samples and a spot urine sample were collected to assess standard biochemical parameters and urinary albumin concentration (UAC, mg/L).

Participants were then instructed to start a 24‐h urine collection. Detailed written and verbal instructions were provided, and patients were encouraged to repeat the collection in case of any doubts or errors. Upon returning the samples, participants were asked to confirm correct collection.

Urine volume of the 24 h was measured in our laboratory. From this, urinary albumin excretion (24 h‐UAE, mg/24 h) and urinary creatinine excretion were measured. A spot urine sample was used to determine urinary albumin concentration (UAC, mg/L) and albumin‐to‐creatinine ratio (ACR, mg/g). Serum and urinary creatinine concentrations were determined using a rate‐blanked compensated Jaffé method traceable to the IDMS reference procedure (CREA Roche/Hitachi automated analysis, Hitachi 917; Roche Diagnostics, Mannheim, Germany). The eGFR was calculated using the Chronic Kidney Disease Epidemiology Collaboration Formula (CKD‐EPI 2021).

### 2.2 Arterial Tonometry

Aortic stiffness was assessed by carotid‐femoral pulse wave velocity (cf‐PWV). Waveforms were recorded at the femoral and carotid sites using SphygmoCor (AtCor Medical, Sydney, NSW, Australia). PWV was calculated from the pulse transit time using the 80% of direct distance method with a rigid sliding calliper, according to the formula: PWV = 0‐.8*d/TT, with d the surface distance between the carotid and the femoral site of measurement. Central haemodynamics was assessed by acquisition of peripheral pressure waveforms of the radial artery at the wrist using applanation tonometry: a generalised validated transfer function (*SphygmoCor software*) was used to generate the corresponding central aortic pressures. Arterial waveforms were calibrated with brachial mean blood pressure and calculated as diastolic blood pressure + 0.4 * pulse pressure. The augmentation index (Aix@75) was calculated as the ratio of augmentation pressure, the difference between the second and first systolic peak, and pulse pressure, expressed as a percentage and adjusted for a heart rate of 75 bpm. An in‐device quality rating of ≥ 80% was required for all recordings. All measurements were performed by trained staff with specific experience in obese individuals, following current international guidelines [17].

### 2.3 Endothelial Function

Methods for the assessment of flow‐mediated dilation (FMD) of the brachial artery, including both traditional and allometric approaches, have been previously described and assessments were conducted in accordance with internationally recognized expert consensus protocols [18]. Briefly, a cuff was placed around the right forearm, and the right brachial artery was identified and scanned longitudinally 5–10 cm above the elbow using a 10‐MHz linear array transducer (MyLab 25; Esaote, Florence, Italy), stabilised by a stereotactic clamp. The cuff was inflated to 300 ± 30 mmHg and deflated after 5 min. Brachial artery diameter and flow velocity were continuously measured using a real‐time computerised edge detection system (Cardiovascular Suite; Quipu srl, Pisa, Italy) for 1 min at baseline, during cuff inflation, and for 4 min following cuff deflation. Endothelium‐independent vasodilation was assessed after the sublingual administration of 25 μg of glyceryl trinitrate (GTN). FMD and response to GTN were calculated as the maximal percentage increase in diameter above baseline.

### 2.4 Renal Resistive Index Measurement

In each participant, three velocity measurements of the interlobular arteries (superior, middle, and inferior) within the meso‐renal region of both kidneys using an ultrasound approach (MyLab 25, Esaote) via the translumbar or anterior method were performed. The Renal Resistive Index (RRI) was computed using the formula: RRI = (peak systolic velocity—end‐diastolic velocity)/peak systolic velocity, where both velocities were measured within the same wave. A single experienced investigator conducted all measurements. The reliability of these measurements by the same observer was evaluated, yielding an intraclass correlation coefficient of 0.95–0.97. The expected variability within these measurements was ≤ ± 3%.

### 2.5 Body Composition Analysis

The resistance and reactance values were determined using a single frequency (0.4 mA, 50 kHz) electrical impedance plethysmograph (EFG–Akern, Firenze, Italy) while patients were in a supine position and fasting. Electrodes were positioned on the dorsal surfaces of the right hand and right foot. Fat free mass (FFM) was computed using manufacturer‐provided equations, utilising the resistance and reactance values along with the patient's height and weight.

### 2.6 Statistical Analysis

Continuous variables were expressed as mean ± standard deviation (SD) or median [interquartile range], depending on their distribution, which was assessed using the Shapiro–Wilk test. Categorical variables were presented as absolute numbers and percentages. Between‐group comparisons were performed using the independent samples *t*‐test or Mann–Whitney *U* test for continuous variables, and the Chi‐square test or Fisher's exact test for categorical variables, as appropriate.

In the calculation of FMD with the allometric scaling approach, baseline and peak brachial diameters were log‐transformed, and the difference between their logarithms, ln(peak‐baseline diameter), was analysed via regression, with ln(baseline diameter) included as a covariate.

Variables with a skewed distribution, including ACR and UAE, were log‐transformed using log(x+1) prior to inclusion in regression models.

Bivariate linear regression models were used to explore the associations between albuminuria markers (UAC, 24 h‐UAE, ACR) and vascular parameters, specifically flow‐mediated dilation (FMD) and carotid‐femoral pulse wave velocity (PWV).

To assess the independent associations between albuminuria markers and vascular outcomes, separate multivariable linear regression models were performed using PWV and allometrically scaled FMD as dependent variables. Each model was adjusted for age, sex, mean blood pressure (MBP), and HbA1c, and included either log‐transformed ACR or log‐transformed 24 h‐UAE as continuous predictors. Multicollinearity was assessed using variance inflation factors (VIFs) for all predictors. All VIF values were < 5, indicating acceptable levels of collinearity.

Unsupervised k‐means clustering analysis (*K* = 2) was performed using four continuous variables to identify distinct vascular‐metabolic phenotypes within the study cohort. Cluster membership was then cross‐tabulated with the presence of type 2 diabetes, hypertension, and smoking status.

Statistical tests were performed using JMP Pro 16.3.0 (SAS Institute Inc., Cary, NC, USA) using a two‐sided *α* level of 0.05.

## 3 Results

### 3.1 General Features of the Study Population

A total of 140 participants (51 males, 89 females) undergoing preoperative evaluation for bariatric surgery were studied (Table 1). Males had significantly higher body weight, body surface area (BSA), and fat‐free mass (FFM) compared to females (*p* < 0.001 for all), although BMI was similar between sex.

**TABLE 1:** Anthropometrics and clinical features of the total cohort of subjects.

	Total cohort (*n* = 140)	Male	Female	*p* value
*n*	140	51	89	
Age (years)	46 ± 10	46 ± 8	47 ± 11	0.776
Weight (kg)	126 ± 25	140 ± 22	118 ± 22	< 0.001
BMI (kg/m^2^)	45.0 ± 6.1	45.6 ± 5.5	44.6 ± 7.4	0.348
BSA (m^2^)	2.28 ± 0.25	2.48 ± 0.22	2.18 ± 0.20	< 0.001
Free fatty mass (kg)	67.4 ± 14.9	84.0 ± 9.9	58.2 ± 7.4	< 0.001
FFM/BW (%)	53.7 ± 7.2	60.1 ± 5.5	50.1 ± 5.4	< 0.001
Smoking, *n* (%)	32 (23)	11 (22)	21 (24)	0.783
T2D, *n* (%)	42 (30)	15 (29)	27 (30)	0.908
Hypertension, *n* (%)	60 (43)	24 (47)	36 (40)	0.447
SBP (mmHg)	138 ± 13	140 ± 11	135 ± 13	0.067
DBP (mmHg)	87 ± 9	87 ± 7	86 ± 9	0.713
MBP (mmHg)	103 ± 8	105 ± 7	102 ± 8	0.148
Heart rate (bpm)	81 ± 10	80 ± 10	82 ± 12	0.583
PP (mmHg)	51 ± 11	53 ± 8	50 ± 12	0.091
eGFR (ml/min/1.73 m^2^)	98 ± 17	100 ± 13	96 ± 16	0.169
UAC (> 20 mg/L), *n* (%)	31 (22)	14 (27)	17 (19)	0.252
24h‐UAE (> 30 mg), *n* (%)	36 (26)	18 (35)	18 (20)	0.049
ACR (> 30 mg/g), *n* (%)	22 (16)	8 (16)	14 (16)	0.994
UAC (mg/L)	0 [0–17]	6 [0–23]	0 [0–9]	0.041
24 h‐UAE (mg)	0 [0–32]	9 [0–42]	0 [0–15]	0.062
ACR (mg/g)	0 [0–13]	4 [0–16]	0 [0–9]	0.199
Urine creatinine (mg/dL)	99 ± 51	132 ± 54	81 ± 39	< 0.001
Urinary creatinine (mg/24h)	1913 ± 495	2398 ± 415	1635 ± 273	< 0.001
Urinary volume 24h (L)	2.26 ± 0.80	2.10 ± 0.81	2.34 ± 0.78	0.106
Glycaemia (mg/dL)	100 ± 16	104 ± 14	98 ± 16	0.299
HbA1c (%)	6.1 ± 0.8	6.2 ± 0.6	6.1 ± 0.7	0.402
Total cholesterol (mg/dL)	183 ± 34	188 ± 35	181 ± 32	0.289
HDL (mg/dL)	49 ± 13	50 ± 12	49 ± 13	0.444
LDL (mg/dL)	111 ± 26	113 ± 26	110 ± 24	0.198
Triglycerides (mg/dL)	141 [108–200]	156 [110–213]	139 [102–192]	0.282

Abbreviations: ACR, albumin‐to‐creatinine ratio; DBP, diastolic blood pressure; PP, pulse pressure; SBP, systolic blood pressure; UAC, urinary albumin concentration; UAE, urinary albumin excretion.

The prevalence of type 2 diabetes (30%) and hypertension (43%) were similar across sexes. Systolic blood pressure was slightly higher in males without reaching statistical significance (*p* = 0.067). Renal function was preserved in the overall cohort, with eGFR: 98 ± 17 mL/min/1.73 m^2^.

With regard to metabolic parameters, fasting glucose and HbA1c, as well as LDL cholesterol, were comparable between the sexes.

As expected, males exhibited markedly higher urinary creatinine concentrations and 24‐h urinary creatinine excretion (*p* < 0.001).

Median values of albuminuria markers were generally low, although males displayed significantly higher 24 h‐UAC values than females (*p* = 0.041). Interestingly, the prevalence of ACR ≥ 30 mg/g was present in 16% of the population with no sex difference (*p* = 0.994), while 24 h‐UAE ≥ 30 mg was higher in males compared to females (35 vs. 20%, *p* = 0.049).

### 3.2 Concordance Between ACR and UAE

As shown in Table 2, only 22 of 140 patients (16%) had both 24 h‐UAE and ACR above the diagnostic thresholds (≥ 30 mg/24 h and ≥ 30 mg/g, respectively). No patient had an elevated ACR with normal 24 h‐UAE. Interestingly, an additional 14 patients (10%) showed elevated 24 h‐UAE but normal ACR.

**TABLE 2:** Concordance between albumin‐to‐creatinine ratio (ACR) and 24‐h urinary albumin excretion (24 h‐UAE) in the study population (*n* = 140).

Total cohort (*n* = 140)	ACR ≥ 30 mg/g	ACR < 30 mg/g
24 h‐UAE ≥ 30 mg	22 (16)	14 (10)
24 h‐UAE < 30 mg	0 (0)	104 (74)

Female (*n* = 89)	ACR ≥ 30 mg/g	ACR < 30 mg/g
24 h‐UAE ≥ 30 mg	14 (16)	4 (4)
24 h‐UAE < 30 mg	0 (0)	71 (80)

Male (*n* = 51)	ACR ≥ 30 mg/g	ACR < 30 mg/g
24 h‐UAE ≥ 30 mg	8 (16)	10 (19)
24 h‐UAE < 30 mg	0 (0)	33 (65)

*Note:* Albuminuria was defined as ACR ≥ 30 mg/g and/or 24 h‐UAE ≥ 30 mg.

This pattern was particularly evident in males, where only 8 of 18 patients with 24 h‐UAE ≥ 30 mg/24 h also had ACR ≥ 30 mg/g, while 10 had elevated 24 h‐UAE despite a ACR< 30 mg/g (55%). In contrast, females showed better concordance, with only 4 out of 18 (22%) having 24 h‐UAE ≥ 30 mg/24 h and ACR < 30 mg/g.

### 3.3 Vascular Findings in the Albuminuria‐Stratified Subgroup

A total of 70 participants underwent a complete vascular evaluation, including cf‐PWV and FMD. These were stratified into three groups: ACR+ (*n* = 22, both ACR and 24 h‐UAE ≥ 30), UAE+ (*n* = 14, 24 h‐UAE ≥ 30 mg but ACR < 30 mg/g), and No‐ALB (*n* = 34, both ACR and 24 h‐UAE < 30) (Table 3).

**TABLE 3:** Main characteristics of participants with complete vascular assessment stratified by albuminuria profile.

	ACR +	UAE +	No‐ALB	*p* value
*n*	22	14	34	
Age (years)	50 ± 9	44 ± 10	46 ± 10	0.172
Sex, F/M	14/8	4/10	17/17	0.122
Weight (kg)	118 ± 20	162 ± 22^b^ ^,^ ^a^	129 ± 21	< 0.001
BMI (kg/m^2^)	42.4 ± 5.9	51.6 ± 5.6^b^ ^,^ ^a^	44.7 ± 4.2	< 0.001
BSA (m^2^)	2.23 ± 0.20	2.66 ± 0.23^b^ ^,^ ^a^	2.34 ± 0.24	< 0.001
Smoking, *n* (%)	10 (45)	7 (50)	6 (18)	0.030
T2D, *n* (%)	12 (55)	4 (33)	6 (20)	0.032
Hypertension, *n* (%)	17 (77)^b^	7 (50)	13 (38)	0.002
aSBP (mmHg)	136 ± 10^b^	134 ± 13	131 ± 14	0.029
aDBP(mmHg)	86 ± 12	84 ± 10	82 ± 10	0.066
aMBP (mmHg)	105 ± 6^b^	103 ± 11	99 ± 9	0.017
eGFR (ml/min/1.73 m^2^)	91 ± 16	107 ± 18^b^ ^,^ ^a^	100 ± 15°	0.039
UAC (mg/L)	48 [25–89]^b^	27 [19–35]^a^ ^,^ ^b^	5 [2–8]	< 0.001
24h‐UAE (mg)	88 [69–143]^b^	41 [36–48]^a^ ^,^ ^b^	9 [0–12]	< 0.001
ACR (mg/g)	45 [39–87]^b^	18 [15–21]^a^ ^,^ ^b^	4 [0–6]	< 0.001
Urine creatinine (mg/dL)	93 ± 48	154 ± 54^a^ ^,^ ^b^	121 ± 52	0.005
Urinary creatinine (mg/24h)	1743 ± 406^b^	2490 ± 569^a^ ^,^ ^b^	1995 ± 487	< 0.001
Glycaemia (mg/dL)	119 ± 21^b^	109 ± 16	95 ± 11	0.037
HbA1c (%)	6.9 ± 1.1	6.5 ± 0.7^b^	6.2 ± 0.8^a^	0.007
LDL (mg/dL)	110 ± 28	114 ± 25	112 ± 21	0.410
Triglycerides (mg/dL)	161 [112–231]	156 [109–215]	149 [100–202]	0.199
Aix@75 (%)	23.5 ± 11.6	25.1 ± 10.9	26.3 ± 12.3	0.297
Cf‐PWV (m/s)	8.44 ± 1.00^b^	8.18 ± 1.05	7.61 ± 1.12	0.016
Brachial artery diameter (mm)	4.09 ± 0.36	4.28 ± 0.32^a^	4.24 ± 0.32^a^	0.049
FMD (%), traditional	4.43 ± 1.11^b^	4.64 ± 1.88^b^	5.94 ± 2.30	0.017
FMD (%), allometric scaling	4.32 ± 0.61^b^	4.66 ± 0.81^b^	6.09 ± 0.98	0.011
Response to GTN (%)	6.42 ± 1.56	6.69 ± 1.89	7.14 ± 2.22	0.298
RRI	0.68 ± 0.05	0.65 ± 0.05	0.64 ± 0.04	0.123

*Note:* ACR + includes individuals with both ACR ≥ 30 mg/g and 24 h‐UAE ≥ 30 mg. UAE + includes those with 24 h‐UAE ≥ 30 mg but ACR < 30 mg/g. No‐ALB refers to participants with both ACR < 30 mg/g and 24 h‐UAE < 30 mg.

^a^ < 0.05 vs. ACR^.^

^b^ < 0.05 vs. No‐ALB.

Abbreviations: ACR, albumin‐to‐creatinine ratio; aDBP, aortic diastolic blood pressure; Aix@75, augmentation index normalised to a heart rate of 75 beats per minute; aMBP, aortic mean blood pressure; aSBP, aortic systolic blood pressure; BSA, body surface area; cf‐PWV, carotid‐femoral pulse wave velocity; FMD, flow‐mediated dilation; GTN, vasodilation after glyceryl trinitrate.UAC, urinary albumin concentration; UAE, urinary albumin excretion.

The ACR+ group had a higher prevalence of hypertension (77%) and worse glycaemic control compared to both groups.

The UAE+ group displayed significantly greater body weight, BMI, and BSA compared to both ACR+ and No‐ALB groups, along with higher urinary creatinine excretion.

**FIGURE 1:**
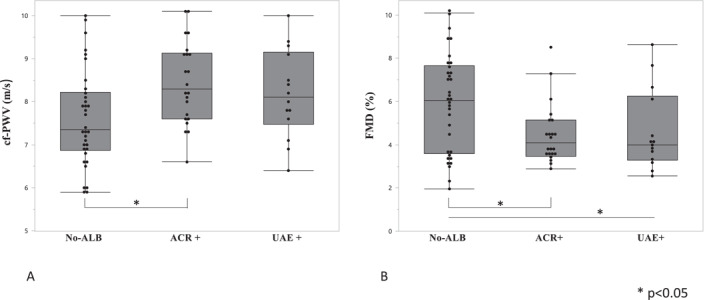
Comparison of vascular parameters across albuminuria subgroups. (A) Carotid–femoral pulse wave velocity (cf‐PWV) and (B) Flow‐mediated dilation (FMD). Boxes represent median and interquartile ranges; whiskers indicate the fifth–95th percentiles. Group comparisons assessed by Kruskal–Wallis test with post hoc Dunn correction.

Regarding vascular parameters, cf‐PWV was significantly increased in ACR + patients compared with No‐ALB (8.44 ± 1.00 vs. 7.61 ± 1.12 m/s) (Figure 1A). The augmented index (AIx@75) did not differ between groups. As expected, both ACR+ and UAE+ groups showed significantly lower FMD values compared to No‐ALB using both traditional and allometric scaling methods (*p* = 0.009 and *p* = 0.003, respectively) (Figure 1B).

### 3.4 Multivariable Regression Analysis

In a multivariable linear model adjusted for age, sex, and BMI, fat‐free mass (FFM) was the strongest independent determinant of 24‐h urinary creatinine excretion (st.*β* = 0.50, *p* < 0.001). Female sex was associated with lower creatinine excretion (*β* = −0.37, *p* = 0.001), and a significant interaction was observed between FFM and sex (*p* = 0.017) (Figure S1).

Two separate multivariable linear models were performed using cf‐PWV as the dependent variable to assess its association with albuminuria markers. These models were restricted to a subgroup of 70 participants who underwent vascular assessment, as described in the Methods section.

In the first model, including log‐transformed ACR, the association with cf‐PWV was statistically significant (st.*β* = 0.21, *p* = 0.045) after adjustment for age, sex, mean blood pressure and HbA1c. In this model, age (*p* = 0.001), HbA1c (*p* = 0.003), and male sex (*p* = 0.032) were also independently associated with higher cf‐PWV. In the second model with log‐transformed 24h‐UAE, the association with PWV showed a trend (st.*β* = 0.19, *p* = 0.061), while age (*p* = 0.004) and HbA1c (*p* = 0.002), but not sex, were significant predictors.

**FIGURE 2:**
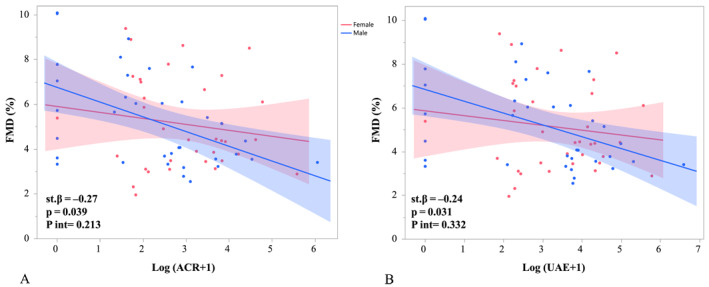
Association between albuminuria markers and allometrically scaled flow‐mediated dilation (FMD) stratified by sex. (a) Log‐transformed ACR showed a significant inverse association with FMD in adjusted multivariable analysis. No significant interaction with sex was found. (b) Log‐transformed 24h‐UAE showed a comparable inverse association with FMD with no significant sex interaction. The models were adjusted for age, sex, mean blood pressure, and HbA1c. Regression lines and 95% confidence intervals are shown separately for males (red) and females (blue).

To evaluate the relationship between albuminuria and endothelial function, two multivariable models were built using allometrically scaled FMD as the dependent variable, adjusting for age, sex, mean blood pressure, and HbA1c. In the first model, log‐transformed ACR was significantly associated with lower FMD (st.*β* = −0.27, *p* = 0.039) (Figure 2a). To assess whether this association differed by sex, a separate simplified model including an interaction term (log[ACR+1] × sex) was tested. The interaction was not statistically significant (*p* = 0.213).

In the second model, log‐transformed 24h‐UAE also showed a significant inverse association with FMD (st.*β* = −0.24, *p* = 0.031) (Figure 2b). Similarly, an interaction term between log[24 h‐UAE+1] and sex was not significant (*p* = 0.332), suggesting consistent effects across sexes in both models.

Finally, to further explore whether vascular and metabolic parameters aggregated into distinct phenotypic patterns, we performed an unsupervised K‐means clustering analysis (*K* = 2) including 24 h‐ UAE, cf‐PWV, FMD, and FFM. This identified two clearly distinct groups: Cluster 1, characterised by preserved vascular parameters, and Cluster 2, defined by high arterial stiffness, low endothelial function and elevated albuminuria. T2D, hypertension, and smoking were significantly more frequent in Cluster 2 (Table S1). These findings support the notion of a clinically relevant high‐risk phenotype, independent of sex.

## 4 Discussion

In this study of individuals with obesity undergoing bariatric surgery work‐up, we found a notable discrepancy in albuminuria classification between ACR and 24 h‐UAE, with ACR failing to detect a substantial proportion of albuminuric participants, particularly males, who were instead identified through the measure of 24‐h albumin excretion. In fact, 24 h‐UAE ≥ 30 mg was more frequent in males, whereas ACR ≥ 30 mg/g showed no sex difference.

Under steady‐state conditions, urinary creatinine excretion reflects creatinine production, which mainly derives from the irreversible conversion of creatine and phosphocreatine in skeletal muscle. Since about 98% of total body creatine is stored in muscle, creatinine excretion is strongly dependent on muscle mass [19, 20].

In a population‐based study of 2.711 individuals, Abdelmalek et al. demonstrated that the creatinine excretion rate increases with body weight, reporting 22% and 46% higher values in the middle and upper BMI tertiles (mean BMI 25.5 and 29.3 kg/m^2^, respectively) compared to the lowest tertile (mean BMI 22.8 kg/m^2^). Similar findings were observed by Ogna et al. in a large cohort of healthy Swiss adults [21]. Furthermore, in a cohort of individuals with severe obesity, Donadio et al. showed that 24‐h urinary creatinine excretion was substantially higher in males than in females, with median values of 2.433 mg/24 h and 1.428 mg/24 h, respectively, aligning with the sex‐related differences observed in the present study [22].

Moving from this evidence, the discordance between ACR and UAE observed in our study appeared to be primarily driven by differences in fat‐free mass, which was significantly higher in males compared to females, despite similar BMI values. In fact, although BMI is commonly used for obesity screening, it is not a direct measure of body composition and does not distinguish between fat and lean mass. As such, individuals with similar BMI may have substantially different levels of FFM, particularly across sexes, limiting its ability to capture physiologic differences relevant to creatinine metabolism [23].

Indeed, multivariable analysis confirmed that FFM was the strongest determinant of 24‐h urinary creatinine, with a significant interaction with sex, suggesting that the relationship between FFM and creatinine output differs by sex, with the effect of FFM appearing less pronounced in females.

This attenuation may reflect known physiological differences in muscle composition and metabolism between sexes. For a given amount of lean mass, males tend to exhibit greater creatinine production, likely due to a higher proportion of type II muscle fibres, increased phosphocreatine content, and the influence of androgens on muscle metabolism [24, 25, 26].

The mechanism highlighted in the present study, whereby any increase in creatinine excretion leads to an underestimation of albuminuria when assessed using ACR, has been demonstrated in large‐scale studies. Notably, in the Chronic Renal Insufficiency Cohort (CRIC) study, ACR bias shifted from a mild overestimation in lean women (+15%) to a significant underestimation (−15%) in those with class III obesity. Among men, where ACR already underestimated albuminuria in lean individuals (−21%), and the bias became even more pronounced with increasing BMI, reaching approximately 40% in class II–III obesity [27].

Importantly, our vascular analyses showed that UAE+ patients (with discordant ACR) had similar levels of vascular impairment, in terms of reduced FMD, as those with concordantly elevated ACR and 24h‐UAE. Both groups exhibited significantly lower endothelial function compared to normoalbuminuric controls, even after adjusting for age, sex, blood pressure, and glycaemic control, supporting their parallel ability in detecting early endothelial dysfunction.

When evaluating arterial stiffness, ACR was independently associated with higher PWV, whereas 24 h‐UAE showed a significant trend, suggesting that ACR may have a stronger association with early increases in arterial stiffness in this population. As expected, age and HbA1c were consistent predictors of PWV in both models, further highlighting their contribution to vascular remodelling.

In males with severe obesity, greater muscle mass and higher creatinine excretion may delay the detection of albuminuria through ACR, such that only those with more advanced renal or vascular damage eventually exceed the diagnostic threshold. This could explain why, in an observational study on diabetic kidney disease cohorts, which included individuals with obesity (mean BMI 30 ± 5.3 kg/m^2^), ACR was a better predictor of renal progression in men [28]. Supporting this hypothesis, a study by Lu et al. demonstrated that albuminuria (ACR ≥ 30 mg/g) was independently associated with subclinical atherosclerosis in men but not in women, despite similar BMI levels across sex groups [29]. Similarly, in a large case‐control study on overweight individuals with T2D, ACR was significantly associated with ischaemic heart disease in men (OR 2.84, *p* < 0.001), but not in women [30].

Interestingly, a large meta‐analysis by Nitsch et al. reported that an ACR ≥ 30 mg/g was more strongly associated with all‐cause mortality in women than in men [31]. While this suggests a greater prognostic value of albuminuria in females, these findings must be interpreted according to the population characteristics. The cohorts included in that analysis were primarily drawn from the general population, with a predominance of normal‐weight individuals, in whom urinary creatinine excretion tends to be more similar between sexes, resulting in a more consistent performance of the ACR. In contrast, in severe obesity, sex differences in creatinine excretion become more pronounced, potentially leading to systematic underestimation of albuminuria by ACR in men, as observed in our cohort.

Notably, all the aforementioned studies addressing sex differences in albuminuria rarely incorporated direct measures of body composition. As such, the observed disparities are often attributed to biological sex alone, without assessing whether fat‐free mass, rather than sex per se, might be the primary driver of the differential impact of creatinine‐based albuminuria estimates. To date, no studies have specifically addressed the prognostic impact of albuminuria on cardiovascular outcomes in severely obese populations stratified by sex. This remains an important gap in the literature, particularly given the physiological and diagnostic asymmetries observed across sexes in this context.

This study has several limitations. First, the sample size was relatively small, particularly for subgroup analyses, which may limit statistical power and the generalisability of findings. Second, the cross‐sectional design precludes causal inferences regarding the relationship between albuminuria markers and vascular dysfunction. A strength aspect is that the study population consisted exclusively of individuals with severe obesity, providing precious information on such a category, marked by a particularly high cardiovascular risk, and requiring a precise risk stratification.

In conclusion, these findings suggest that in individuals with obesity, particularly in males with higher lean mass, reliance on ACR alone may lead to under recognition of albuminuria‐associated vascular risk. 24 h‐UAE may offer a more accurate reflection of true albumin burden in this population, especially when early vascular changes are a concern.

## Author Contributions

D.M. made substantial contributions to the conception of the study, statistical analysis, carried out the experiment, and was involved in drafting the manuscript. M.N. was involved in drafting the manuscript. M.J. carried out the experiments. A.S. was involved in critically revising the article for important intellectual content. R.M.B. carried out experiments, made substantial contributions to the statistical analysis and interpretation of data, and she revised the article.

## Disclosure

The authors have nothing to report.

## Conflicts of Interest

The authors declare no conflicts of interest.

## Peer Review

The peer review history for this article is available at https://www.webofscience.com/api/gateway/wos/peer-review/10.1002/dmrr.70064.

## Supporting information

Supporting Information S1

Figure S1

Table S1

## Data Availability

The data that support the findings of this study are available from the corresponding author upon reasonable request.
