# Supplementary material for: Albumin‐To‐Creatinine Ratio Underestimates True 24‐Hour Albuminuria in Obesity: Clinical Relevance for Vascular Risk Stratification
Source: Diabetes Metab Res Rev. 2025 Jun 25;41(5):e70064. doi: 10.1002/dmrr.70064 (PMC12188699; doi:10.1002/dmrr.70064)
Supplement: Supplementary file 1 — Supporting Information S1 [file DMRR-41-e70064-s002.docx]

**Figure S1.** Relationship between fat-free mass (FFM) and 24-hour urinary creatinine excretion, stratified by sex. Regression lines and 95% confidence intervals are shown for males (red) and females (blue). While creatinine excretion increased with FFM in both sexes, males consistently exhibited higher values across the entire range of FFM. This sex-related shift reflects both an independent effect of sex and a significant interaction between sex and FFM (p = 0.016), indicating a sex-specific relationship in creatinine generation relative to lean mass.
