# Supplementary figures and images for: Albumin‐To‐Creatinine Ratio Underestimates True 24‐Hour Albuminuria in Obesity: Clinical Relevance for Vascular Risk Stratification
Source: Diabetes Metab Res Rev. 2025 Jun 25;41(5):e70064. doi: 10.1002/dmrr.70064 (PMC12188699; doi:10.1002/dmrr.70064)

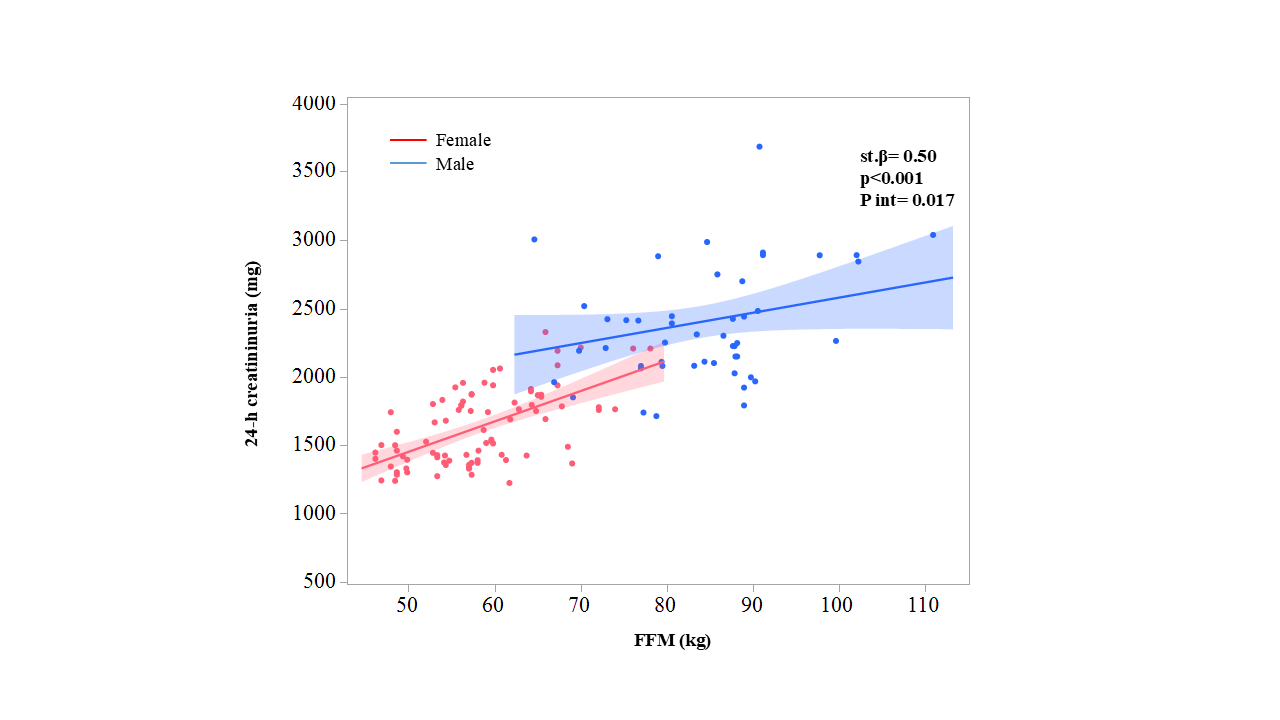

Supplement: Supplementary file 2 — Figure S1 [file DMRR-41-e70064-s003.tif]
