# Supplementary material for: Albumin‐To‐Creatinine Ratio Underestimates True 24‐Hour Albuminuria in Obesity: Clinical Relevance for Vascular Risk Stratification
Source: Diabetes Metab Res Rev. 2025 Jun 25;41(5):e70064. doi: 10.1002/dmrr.70064 (PMC12188699; doi:10.1002/dmrr.70064)
Supplement: Supplementary file 3 — Table S1 [file DMRR-41-e70064-s001.docx]

**Supplementary Table S1. Clinical characteristics of the two clusters derived from unsupervised K-means analysis.**

Clusters were identified based on log-transformed 24-hour urinary albumin excretion (UAE), fat-free mass (FFM), carotid-femoral pulse wave velocity (cf-PWV), and flow-mediated dilation (FMD). Values are presented as means or percentages.

| **Variable** | **Cluster 1 (n=32)** | **Cluster 2 (n=38)** | **p-value** |
| --- | --- | --- | --- |
| Female sex, n(%) | 16 (50) | 19 (50) | 1.00 |
| cf-PWV (m/s) | 7.13 | 8.92 | <0.001 |
| log(UAE+1) | 2.13 | 3.93 | <0.001 |
| FMD (%) | 6.88 | 3.81 | <0.001 |
| FFM (kg) | 71.6 | 73.6 | 0.431 |
| Diabetes, n(%) | 6 (19) | 19 (50) | <0.001 |
| Hypertension, n(%) | 7 (22) | 30 (79) | <0.001 |
| Smoking, n(%) | 5 (16) | 18 (47) | <0.001 |
